# A Short-Term Model of Colitis-Associated Colorectal Cancer That Suggests Initial Tumor Development and the Characteristics of Cancer Stem Cells

**DOI:** 10.3390/ijms241411697

**Published:** 2023-07-20

**Authors:** Yasushi Matsumoto, Toshiro Fukui, Shunsuke Horitani, Yuji Tanimura, Ryo Suzuki, Takashi Tomiyama, Yusuke Honzawa, Tomomitsu Tahara, Kazuichi Okazaki, Makoto Naganuma

**Affiliations:** Division of Gastroenterology and Hepatology, Third Department of Internal Medicine, Kansai Medical University, Hirakata 573-1010, Japan

**Keywords:** mouse model, colitis-associated colorectal cancer, carcinogenesis, cancer stem cell, Smad

## Abstract

The mechanisms underlying the transition from colitis-associated inflammation to carcinogenesis and the cell origin of cancer formation are still unclear. The azoxymethane (AOM)/dextran sodium sulfate (DSS) mouse model reproduces human colitis-associated colorectal cancer. To elucidate the mechanisms of cancer development and dynamics of the linker threonine-phosphorylated Smad2/3 (pSmad2/3L-Thr)-positive cells, we explored the early stages of colitis-associated colorectal cancer in AOM/DSS mice. The AOM/DSS mice were sacrificed at 4 to 6 weeks following AOM administration. To analyze the initial lesions, immunofluorescence staining for the following markers was performed: β-catenin, Ki67, CDK4, Sox9, Bmi1, cyclin D1, and pSmad2/3L-Thr. Micro-neoplastic lesions were flat and unrecognizable, and the uni-cryptal ones were either open to the surfaces or hidden within the mucosae. These neoplastic cells overexpressed β-catenin, Sox9, Ki67, and Cyclin D1 and had large basophilic nuclei in the immature atypical cells. In both the lesions, pSmad2/3L-Thr-positive cells were scattered and showed immunohistochemical co-localization with β-catenin, CDK4, and Bmi1 but never with Ki67. More β-catenin-positive neoplastic cells of both lesions were detected at the top compared to the base or center of the mucosae. We confirmed initial lesions in the colitis-associated colorectal cancer model mice and observed results that suggest that pSmad2/3L-Thr is a biomarker for tissue stem cells and cancer stem cells.

## 1. Introduction

Ulcerative colitis (UC) increases the risk of developing colorectal cancer (CRC) [1]; the number of UC-associated CRC patients is increasing worldwide as the number of UC patients increases [2]. The risk of developing UC-associated CRC depends on the extent and duration of inflammation of UC and genetic predisposition [3]. Nevertheless, the detailed mechanism underlying the transition from UC-associated inflammation to carcinogenesis remains to be elucidated.

Several animal models of colitis-associated CRC have been developed in rodents. The induction of the best-studied mouse model of chemical-induced colitis-associated CRC requires a single intraperitoneal injection of the colon carcinogen azoxymethane (AOM) followed by colitis induction through the oral administration of dextran sodium sulfate (DSS) [4,5]. This AOM/DSS mouse model recapitulates the course of human colitis-associated CRC from inflammation to dysplasia and cancer, causing severe colitis with weight loss, bloody diarrhea, and multiple colon tumors [5,6].

Recently, the cancer stem cell (CSC) theory has been accepted as an intriguing hypothesis for cancer development and progression. The theory suggests that cancers are composed of functionally heterogenous subsets of cells. In the CSC model, one small subset of cancer cells has stem cell characteristics. These CSCs have both self-renewal capacity and the ability to differentiate into various cancer cells and play an important role in maintaining the growth, invasion, metastasis, and recurrence capacity of malignant tumors [7,8]. Given that CSCs are relatively resistant to therapies developed to eradicate the non-CSC component of cancer, the CSC model provides a theoretical basis for developing therapeutic strategies targeting a minority of CSC populations and presents new perspectives on cancer treatment [8].

Although CRC has been thoroughly studied, the cell of origin for carcinogenesis is still poorly understood. Two hypotheses have been proposed: the bottom-up model and the top-down model. The bottom-up model proposes that normal stem cells are the first transformed cells, either directly giving rise to cancer cells or reprograming themselves to acquire CSC behavior before inducing cancer [9,10]. However, histological evidence hints at a top-down model of CRC development, in which CRC can also arise from late progenitors or early differentiated cells. The top-down model is supported by the finding that dysplastic cells are routinely found at the luminal surface of the crypts, while the cells at the bases of these same crypts appear morphologically normal during the early stages of carcinogenesis [11]. Additionally, aberrant crypt foci (ACF), putative precursor lesions for CRC, in AOM (and DSS)-treated rodents are observed by superficial examination of the luminal mucosa of methylene blue-stained colon preparations [12,13,14]. On the other hand, Schwitalla et al. have suggested that these theories may not necessarily be mutually exclusive and that tumor-initiating mutations can occur in both normal stem cells and more differentiated cells as long as both cells dedifferentiate and regain stem cell properties through chronic inflammatory signaling [15]. They have demonstrated that epithelial non-stem cells can re-express stem cell markers and be converted into CSCs, providing an additional explanation as to why UC patients are at increased risk of developing CRC.

Smad proteins are core mediators that transduce signals from transforming growth factor (TGF)-β superfamily receptors to the nuclei. They are regulatory proteins composed of conserved Mad homology (MH) 1, intermediate linker, and MH2 domains [16,17]. The catalytic TGF-β type I receptor (TβRI) phosphorylates COOH-terminal serine (Ser) residues of receptor-activated Smads, such as Smad2 and Smad3 [18]. Specific Ser or threonine (Thr) residues within the linker are phosphorylated by Ras-related (proline-directed) kinases, which consist of extracellular signal-regulated kinase (ERK), c-Jun NH2-terminal kinase (JNK), and cyclin-dependent kinase (CDK) 4 [19,20,21,22]. TβRI and Ras-related kinases, specifically phosphorylate Smad2 and Smad3, generate several phosphoisoforms: Smad2/3 are phosphorylated at the COOH-terminal (pSmad2C and pSmad3C); Smad2/3 are phosphorylated at the linker (pSmad2L and pSmad3L); and Smad2/3 are phosphorylated at both the C-terminal and linker (pSmad2C/L and pSmad3C/L) [22,23,24,25]. Phosphorylated Smad2 and Smad3 rapidly oligomerize with Smad4 and translocate to the nucleus, where they regulate the transcription of the target genes [26].

In our previous study, we confirmed the specific expression of linker Thr-phosphorylated Smad2/3 (pSmad2/3L-Thr) in mouse colon epithelial cells, suggesting that these cells are colon epithelial stem-like cells [27]. Subsequently, by investigating the AOM/DSS mouse model and examining the Smad2/3 phosphorylation profiles, we clarified that carcinogenic pSmad3L-Ser signaling triggered by chronic colitis is a key early event in colitis-associated CRC. Furthermore, the study supported the theory that pSmad2/3L-Thr immunostaining-positive cells are CSCs [28]. In our most recent study, the AOM/DSS mice were sacrificed 10, 20, and 30 weeks following AOM administration [29]. In mice 10 or 20 weeks after AOM administration, most colon tumors showed features of intramucosal adenocarcinoma. In mice 30 weeks after AOM administration, the main lesions further increased in size, and infiltration into the submucosa and vascular invasion, which are considered to be characteristic of early metastatic lesions, were observed at a high rate.

Therefore, in this study, in order to analyze the mechanism of colitis-associated CRC at the time of initial tumor (dysplasia) development, the lesions of AOM/DSS mice 4 to 6 weeks following the administration of AOM were observed in detail, and we intended to confirm the roles of pSmad2/3L-Thr-positive cells as CSCs.

## 2. Results

### 2.1. Immunofluorescence Staining for β-Catenin, Sox9, Ki67, Cyclin D1, or CDK4 and H&E Staining in the Micro-Neoplastic Lesions of the AOM/DSS Mice

The micro-neoplastic lesions (less than 300 μm wide) were flat and not macroscopically recognizable on the mucosal surface in the colons of the AOM/DSS mice 4 to 6 weeks following the administration of AOM.

**Figure 1:**
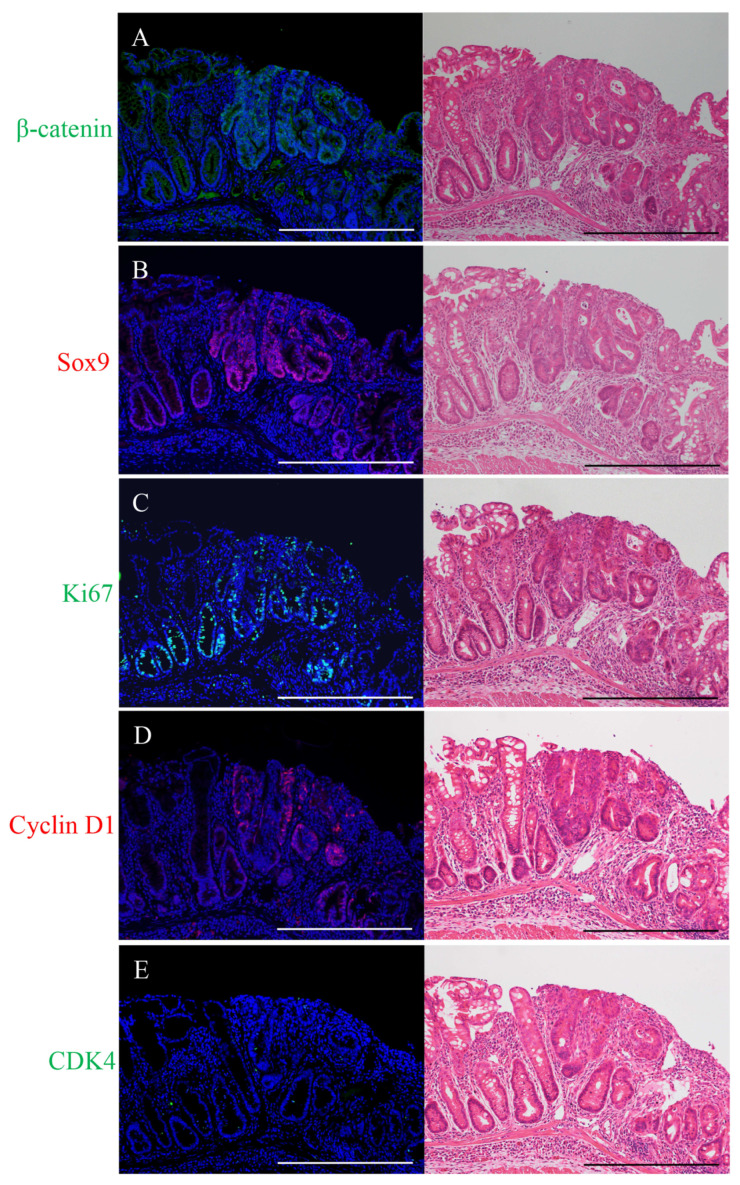
Immunofluorescence staining for (**A**) β-catenin (green), (**B**) Sox9 (red), (**C**) Ki67 (green), (**D**) cyclin D1 (red), and (**E**) CDK4 (green) in the micro-neoplastic lesions of the AOM/DSS mice. DAPI (blue) was used for nuclear staining. (**A**–**E**) In the right panels, the same sections were stained with hematoxylin and eosin after immunofluorescence staining and observed by light microscopy. Original magnification, ×200 (**A**–**E**). Scale bars: 300 μm (**A**–**E**).

Immunostaining-positive cells of β-catenin (green; Figure 1A), Sox9 (red; Figure 1B), Ki67 (green; Figure 1C), cyclin D1 (red; Figure 1D), or CDK4 (green; Figure 1E) were observed in the micro-neoplastic lesions of the AOM/DSS mice using DAPI nuclear staining (blue). After immunofluorescence staining, we stained the same sections with H&E and confirmed the lesions and immunostaining-positive cells using a light microscope (right panels in Figure 1).

Immunofluorescence staining for β-catenin showed positivity in the cell membrane of the non-neoplastic epithelia. In the micro-neoplastic lesions, β-catenin-positive cells were distributed throughout the lesions, and their expression was observed predominantly in the cytoplasm and nucleus of the neoplastic cells (Figure 1A).

Sox9-positive cells were detected and confined around the crypt bases of the non-neoplastic epithelia. In the micro-neoplastic lesions, Sox9-positive cells were diffusely distributed throughout the lesions. Their expression was observed in the nucleus of the non-neoplastic and neoplastic cells (Figure 1B).

Ki67-positive cells were detected and confined around the crypt bases of the non-neoplastic epithelia. In the micro-neoplastic lesions, Ki67-positive cells were diffusely scattered throughout the lesions. Their expression was observed in the nucleus of the non-neoplastic and neoplastic cells (Figure 1C).

Immunofluorescence staining for cyclin D1 showed no positive cells in the non-neoplastic epithelia. In the micro-neoplastic lesions, cyclin D1-positive cells were scattered throughout the lesions, and their expression was observed in the nucleus of the neoplastic cells (Figure 1D).

CDK4-positive cells were sparsely detected and confined around the crypt bases of the non-neoplastic epithelia. In the micro-neoplastic lesions, CDK4-positive cells were sparsely scattered in the lesions. Their expression was observed in the nucleus of the non-neoplastic and neoplastic cells (Figure 1E).

The micro-neoplastic lesions showed characteristics of intramucosal adenocarcinoma. The nuclei were enlarged, round, or ovoid, and the nucleoli were prominent; the lesions had tubular structures, but nuclear polarity was significantly lost, with nuclei no longer being oriented perpendicular to the basement membrane; there were numerous mitoses, and goblet cells were almost absent (right panels in Figure 1).

### 2.2. Double Immunofluorescence Staining for pSmad2/3L-Thr with β-Catenin, Ki67, CDK4, or Bmi1 and H&E Staining in the Micro-Neoplastic Lesions of the AOM/DSS Mice

**Figure 2:**
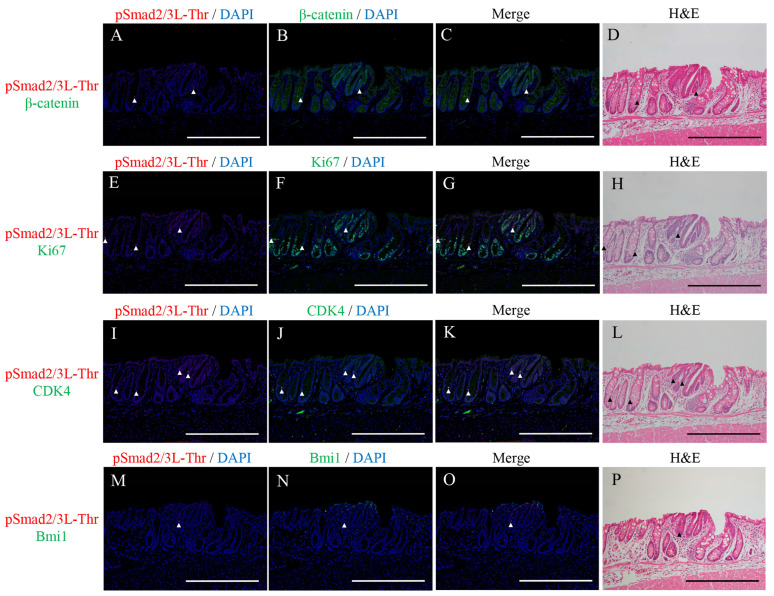
Double immunofluorescence staining for pSmad2/3L-Thr (red; arrowheads) with (**A**–**C**) β-catenin (green), (**E**–**G**) Ki67 (green), (**I**–**K**) CDK4 (green), and (**M**–**O**) Bmi1 (green) in the micro-neoplastic lesions of the AOM/DSS mice. DAPI (blue) was used for nuclear staining. (**D**,**H**,**L**,**P**) The same sections were stained with hematoxylin and eosin after immunofluorescence staining and observed via light microscopy. Original magnification, ×200 (**A**–**P**). Scale bars: 300 μm (**A**–**P**).

Double immunofluorescence staining for pSmad2/3L-Thr (red; arrowheads in Figure 2) with β-catenin (green; Figure 2B,C), Ki67 (green; Figure 2F,G), CDK4 (green; Figure 2J,K), or Bmi1 (green; Figure 2N,O) were performed in the micro-neoplastic lesions of the AOM/DSS mice using DAPI nuclear staining (blue). After immunofluorescence staining, we stained the same sections with H&E and confirmed the lesions and immunostaining-positive cells using a light microscope (Figure 2D,H,L,P).

pSmad2/3L-Thr-positive cells were sparsely detected around the crypt bases of the non-neoplastic epithelia. In the micro-neoplastic lesions, pSmad2/3L-Thr-positive cells were sparsely scattered in the lesions. Their expression was observed in the cytoplasm and nucleus of the non-neoplastic and neoplastic cells (Figure 2A,C,E,G,I,K,M,O).

In both the non-neoplastic and neoplastic cells, pSmad2/3L-Thr-positive cells showed immunohistochemical co-localization with β-catenin (Figure 2C), CDK4 (Figure 2K), and Bmi1 (Figure 2O) but never with Ki67 (Figure 2G).

### 2.3. Locations of the Micro-Neoplastic Lesions in the AOM/DSS Mice within the Mucosae

**Figure 3:**
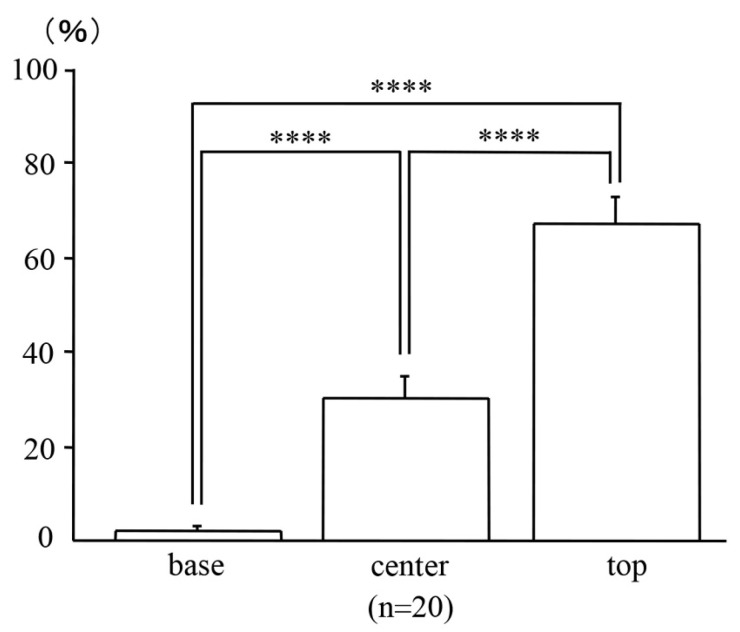
Locations of the micro-neoplastic lesions in the AOM/DSS mice within the mucosae. Bars represent the percentage of locations of the neoplastic cells at the base, center, and top of the mucosae, respectively. Data are expressed as the mean ± standard error of the mean of 20 micro-neoplastic lesions of the AOM/DSS mice. Results were compared using one-way analysis of variance followed by Fisher’s protected least significant difference test (**** represents *p* < 0.0001).

After dividing the mucosae vertically into three sections of the same length, far more β-catenin-positive neoplastic cells of the micro-neoplastic lesions in the AOM/DSS mice were detected at the top of the mucosae (67.33 ± 5.50%) than at the base (2.14 ± 1.03%; *p* < 0.0001) or center (30.18 ± 4.81%; *p* < 0.0001) of the mucosae (Figure 3; *n* = 20).

### 2.4. Immunofluorescence Staining for β-Catenin and H&E Staining in the Uni-, Bi-, or Tri-Cryptal Neoplastic and Micro-Neoplastic Lesions of the AOM/DSS Mice

**Figure 4:**
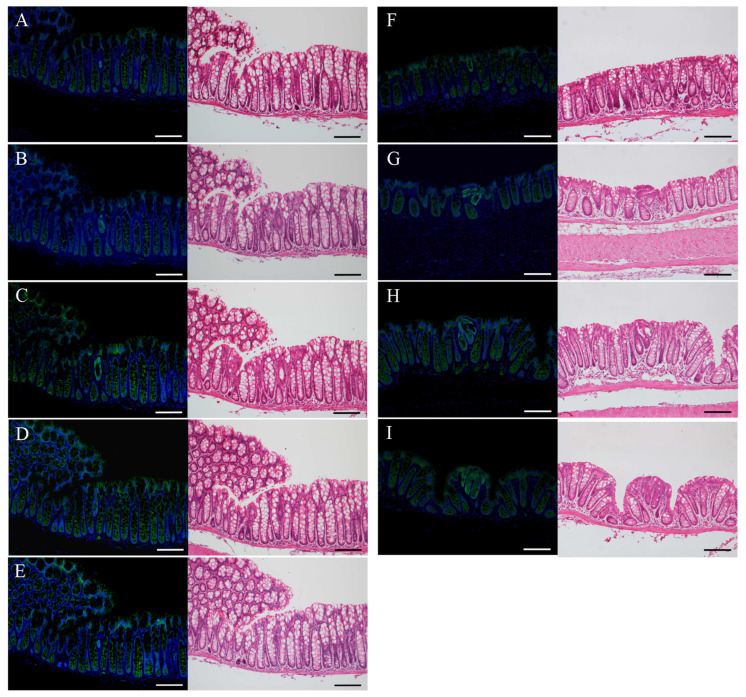
Immunofluorescence staining for β-catenin (green) in the (**A**–**F**) uni-, (**G**) bi-, or (**H**) tri-cryptal neoplastic and (**I**) micro-neoplastic lesions of the AOM/DSS mice. DAPI (blue) was used for nuclear staining. (**A**–**I**) In the right panels, the same sections were stained with hematoxylin and eosin after immunofluorescence staining and observed via light microscopy. (**A**–**E**) Serial sections of the same lesion. Original magnification, ×200 (**A**–**I**). Scale bars: 100 μm (**A**–**I**).

Immunostaining-positive cells of β-catenin (green) were observed in the uni- (Figure 4A–F), bi- (Figure 4G), or tri-cryptal (Figure 4H) neoplastic and micro-neoplastic (Figure 4I) lesions of the AOM/DSS mice using DAPI nuclear staining (blue). After immunofluorescence staining, we stained the same sections with H&E and confirmed the lesions and immunostaining-positive cells using a light microscope (right panels in Figure 4). In the serial sections, the same uni-cryptal neoplastic lesion of the AOM/DSS mice could be completely observed from end to end (Figure 4A–E).

In the uni-, bi-, or tri-cryptal neoplastic and micro-neoplastic lesions, β-catenin-positive cells were distributed throughout the lesions, and their expression was observed predominantly in the cytoplasm and nucleus of the neoplastic cells.

### 2.5. Immunofluorescence Staining for β-Catenin, Sox9, Ki67, Cyclin D1, or CDK4 and H&E Staining in the Uni-Cryptal Neoplastic Lesions of the AOM/DSS Mice

In the uni-cryptal neoplastic lesions of the colons of the AOM/DSS mice 4 to 6 weeks following the administration of AOM, there were either those that opened on the mucosal surfaces or those that were completely hidden within the mucosae without being exposed on the surface.

**Figure 5:**
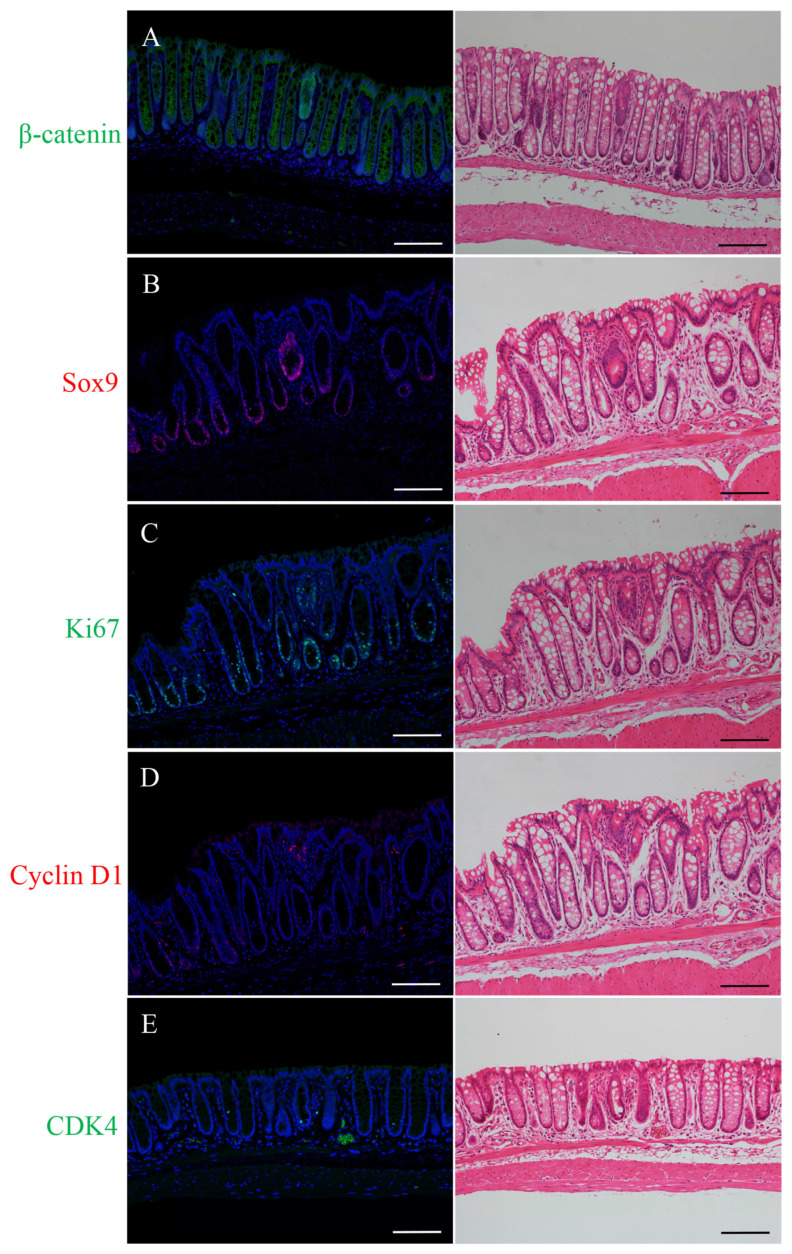
Immunofluorescence staining for (**A**) β-catenin (green), (**B**) Sox9 (red), (**C**) Ki67 (green), (**D**) cyclin D1 (red), and (**E**) CDK4 (green) in the uni-cryptal neoplastic lesions of the AOM/DSS mice. DAPI (blue) was used for nuclear staining. (**A**–**E**) In the right panels, the same sections were stained with hematoxylin and eosin after immunofluorescence staining and observed via light microscopy. Original magnification, ×200 (**A**–**E**). Scale bars: 100 μm (**A**–**E**).

Immunostaining-positive cells of β-catenin (green; Figure 5A), Sox9 (red; Figure 5B), Ki67 (green; Figure 5C), cyclin D1 (red; Figure 5D), or CDK4 (green; Figure 5E) were observed in the uni-cryptal neoplastic lesions of the AOM/DSS mice using DAPI nuclear staining (blue). After immunofluorescence staining, we stained the same sections with H&E and confirmed the lesions and immunostaining-positive cells using a light microscope (right panels in Figure 5).

In the uni-cryptal neoplastic lesions, β-catenin-positive cells (Figure 5A) and Sox9-positive cells (Figure 5B) were distributed throughout the lesions, and their expression was observed in the cytoplasm and nucleus and in the nucleus of the neoplastic cells, respectively. Ki67-positive cells (Figure 5C) and cyclin D1-positive cells (Figure 5D) were scattered throughout the lesions, and their expression was observed in the nucleus of the neoplastic cells. CDK4-positive cells were sparsely scattered in the lesions, and their expression was observed in the nucleus of the neoplastic cells (Figure 5E). These results were precisely the same as those for the micro-neoplastic lesions of the AOM/DSS mice (refer to Figure 1).

### 2.6. Double Immunofluorescence Staining for pSmad2/3L-Thr with β-Catenin, Ki67, CDK4, or Bmi1 and H&E Staining in the Uni-Cryptal Neoplastic Lesions of the AOM/DSS Mice

**Figure 6:**
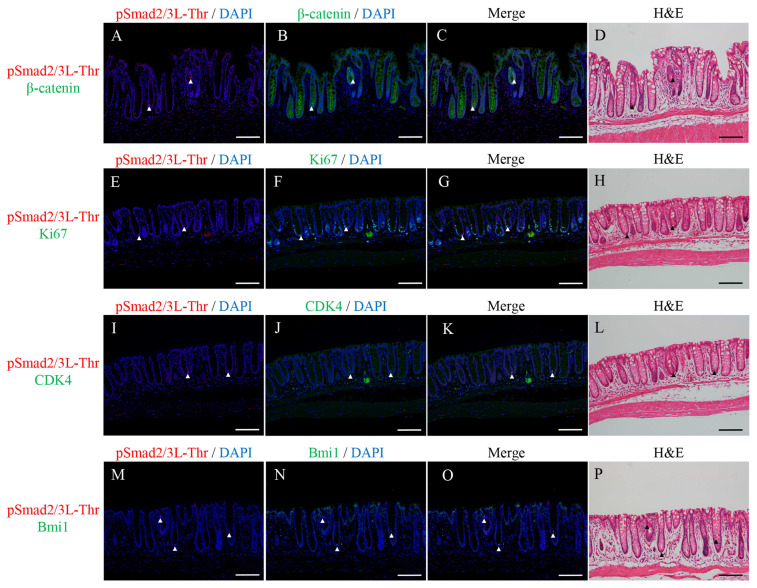
Double immunofluorescence staining for pSmad2/3L-Thr (red; arrowheads) with (**A**–**C**) β-catenin (green), (**E**–**G**) Ki67 (green), (**I**–**K**) CDK4 (green), and (**M**–**O**) Bmi1 (green) in the uni-cryptal neoplastic lesions of the AOM/DSS mice. DAPI (blue) was used for nuclear staining. (**D**,**H**,**L**,**P**) The same sections were stained with hematoxylin and eosin after immunofluorescence staining and observed via light microscopy. Original magnification, ×200 (**A**–**P**). Scale bars: 100 μm (**A**–**P**).

Double immunofluorescence staining for pSmad2/3L-Thr (red; arrowheads in Figure 6) with β-catenin (green; Figure 6B,C), Ki67 (green; Figure 6F,G), CDK4 (green; Figure 6J,K), or Bmi1 (green; Figure 6N,O) was performed in the uni-cryptal neoplastic lesions of the AOM/DSS mice using DAPI nuclear staining (blue). After immunofluorescence staining, we stained the same sections with H&E and confirmed the lesions and immunostaining-positive cells using a light microscope (Figure 6D,H,L,P).

pSmad2/3L-Thr-positive cells were sparsely detected around the crypt bases of the non-neoplastic epithelia. In the uni-cryptal neoplastic lesions, pSmad2/3L-Thr-positive cells were sparsely scattered in the lesions. Their expression was observed in the cytoplasm and nucleus of the non-neoplastic and neoplastic cells (Figure 6A,C,E,G,I,K,M,O).

In both the non-neoplastic and neoplastic cells, pSmad2/3L-Thr-positive cells showed immunohistochemical co-localization with β-catenin (Figure 6C), CDK4 (Figure 6K), and Bmi1 (Figure 6O) but never with Ki67 (Figure 6G).

These results were precisely the same as those for the micro-neoplastic lesions of the AOM/DSS mice (refer to Figure 2).

### 2.7. Locations of the Uni-Cryptal Neoplastic Lesions in the AOM/DSS Mice within the Mucosae

**Figure 7:**
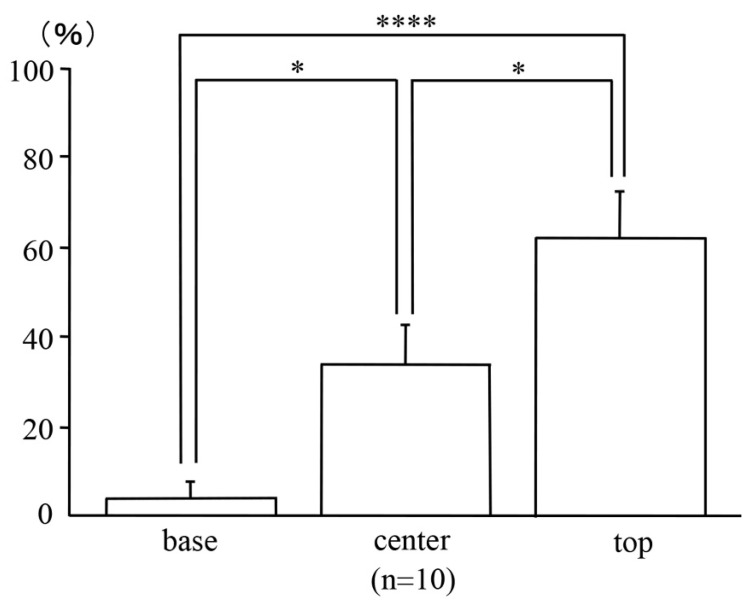
Locations of the uni-cryptal neoplastic lesions in the AOM/DSS mice within the mucosae. Bars represent the percentage of locations of neoplastic cells at the base, center, and top of the mucosae, respectively. Data are expressed as the mean ± standard error of the mean of 10 uni-cryptal neoplastic lesions of the AOM/DSS mice. Results were compared using one-way analysis of variance followed by Fisher’s protected least significant difference test (* and **** represent *p* < 0.05 and *p* < 0.0001, respectively).

To confirm the locations of the uni-cryptal neoplastic lesions within the mucosae, we analyzed those that could be observed completely from end to end on serial sections. After dividing the mucosae vertically into three sections of the same length, far more β-catenin-positive neoplastic cells of the uni-cryptal neoplastic lesions in the AOM/DSS mice were detected at the top of the mucosae (62.49 ± 10.68%) than at the base (3.70 ± 3.70%; *p* < 0.0001) or center (34.01 ± 8.78%; *p* < 0.05) of the mucosae (Figure 7; *n* = 10).

## 3. Discussion

Several animal models that resemble the characteristics of colitis-associated CRC have been reported. The time-honored mouse model uses DSS. However, the development of CRC in the DSS colitis model requires long-term exposure and the frequent administration of DSS, and the incidence and multiplicity of induced neoplasms are low [30,31]. A number of studies have shown that chronic or recurrent mucosal inflammation can cause carcinogenesis through several proposed mechanisms, including the induction of genetic mutations, increased proliferation, altered metabolism, and altered bacterial flora [3]. On the other hand, in the AOM/DSS mouse model, mice injected with a low dose of AOM develop many neoplasms after relatively short-term DSS exposure. Therefore, the AOM/DSS mouse model is suitable for studying colitis-associated CRC, and these dysplasia and neoplasms show positive staining for β-catenin [6].

ACF are early-appearing lesions found on the colonic luminal surface of AOM (and DSS)-treated rodents and UC patients [12,32,33]. ACF are characterized by crypts with altered luminal openings and thickened epithelia that are larger than the adjacent normal crypts [33]. Although the numbers of ACF increase over time after exposure to the carcinogen, evidence correlating the development of neoplasia with ACF expression is weak. Furthermore, in rodents with a predisposition to developing CRC, histological sections (horizontal cross-sections) reveal dysplastic crypts with excessive β-catenin accumulation, termed β-catenin-accumulated crypts (BCAC). BCAC have disrupted cell morphology and cause greater dysplasia than ACF; they increase with time after carcinogen treatment. BCAC are not similar to ACF in terms of appearance and are usually not recognizable on the mucosal surface [34]. However, it is not clear whether BCAC represent a subgroup of ACF or if they can be depicted as a separate entity. These foci spread over several crypts, probably through crypt fission or cell migration [6].

The present study aimed to clarify the mechanism of colitis-associated CRC development and the dynamics of pSmad2/3L-Thr-positive cells by analyzing the very early stages of colitis-associated CRC development in AOM/DSS mice. We initially assumed the veracity and suitability of the top-down model based on our past studies and the present results of the locations of early neoplastic lesions within the mucosae [28,29]. However, lesions coexisting with normal epithelial cells in the same crypts were never observed not only in multi-cryptal neoplastic but also in uni-cryptal neoplastic lesions. In other words, all cells in the uni-cryptal neoplastic lesions of the AOM/DSS mice were dysplastic cells with a high β-catenin expression. From the results of the present study, in the initial neoplastic lesions that occurred in the AOM/DSS mouse model, CSCs mutated from normal tissue stem cells first appeared at the upper site of the mucosae similar to the bottom-up model and formed uni-cryptal neoplastic lesions which grew to bi- and tri-cryptal lesions through crypt fission or cell migration. This is considered to be the same reason that mucosal regeneration starts in the upper site of the mucosae in active colitis in DSS mouse models and human UC [35,36].

Although BCAC were observed only in the horizontal cross-section [34], we made continuous vertical cross-sections in which small neoplastic lesions could be observed from end to end. Similar to the large CRCs in the AOM/DSS mice we previously reported on [28], these neoplastic cells overexpress β-catenin, Sox9, Ki67, and Cyclin D1 and have large basophilic nuclei in the immature atypical cells. BCAC might have been observed in the longitudinal direction.

Our previous studies have confirmed the significant expression of pSmad2/3L-Thr in the normal colon epithelial cells of wild-type mice and the CRCs of AOM/DSS mice, indicating that these cells are colon epithelial stem-like cells and colorectal CSCs, respectively [27,28,29]. Furthermore, we have found that pSmad2/3L-Thr-positive cells retain BrdU labeling, are slow-cycling, and are Ki67-negative resting cells in the G0 phase, located adjacent to the actively proliferating cells of the normal colon epithelial cells [27]. We have consistently believed that pSmad2/3L-Thr identifies normal epithelial stem-like cells in the esophagus, stomach, small intestine, and colon and in colorectal CSCs just before re-entering the cell cycle from the G0 phase (also known as the resting phase) [27,28,29,37,38]. We also performed double immunofluorescence staining for pSmad2/3L-Thr and Bmi1, a representative marker for slow-cycling (cancer) stem cells [39,40]. In the present study, pSmad2/3L-Thr-positive cells were confirmed to be cells with the same site and stainability (Ki67-negative, CDK4- and Bmi1-positive), as previously reported in the surrounding non-neoplastic epithelium and initial neoplastic lesions of the AOM/DSS mice. We were able to reaffirm the results supporting the notion that pSmad2/3L-Thr is a biomarker for normal tissue stem cells and CSCs.

In conclusion, we have confirmed the initial neoplastic lesions in a colitis-associated CRC mice model. Similar changes may be observed in the development of human UC-associated CRC, and we are going to investigate the clinical specimens of patients with UC. As in our previous studies, the present study has shown consistent results that indicate that pSmad2/3L-Thr is a biomarker for tissue stem cells and CSCs.

## 4. Materials and Methods

### 4.1. Mice

Five-week-old male Crl:CD-1 (ICR) mice were purchased from Charles River Laboratories (Charles River Laboratories Japan, Inc., Yokohama, Japan). All the mice were housed in a specific pathogen-free environment within the animal facility of Kansai Medical University. They were given commercial food pellets (F2; Funabashi Farm, Chiba, Japan) and tap water. All of our experimental protocols were approved by the Ethics Committee for the Use of Experimental Animals of Kansai Medical University (Approval Number 23-001).

### 4.2. Chemicals

AOM, a colon carcinogen (Sigma-Aldrich Japan K.K., Tokyo, Japan), and DSS, with a molecular weight of 36,000–50,000 (MP Biomedicals, Solon, OH, USA) were purchased from Sigma-Aldric and MP Biomedicals, respectively. DSS was diluted with water to form a 2% solution to induce colitis.

### 4.3. Experimental Design

A single intraperitoneal injection of AOM (10 mg/kg body weight) was administered to the ICR mice, and one week after this administration, the mice were given 2% DSS in drinking water for 7 days. The mice that received AOM/DSS were sacrificed by cervical dislocation 4 to 6 weeks following AOM administration [5,28,41].

After flushing the lumens with saline, their colons were excised and cut open longitudinally. After washing several times with saline, they were cut and fixed in 10% buffered formalin. Paraffin-embedded sections were prepared using a standard method.

### 4.4. Histopathological Analysis

First, immunofluorescence staining for β-catenin was performed on sections selected at appropriate intervals to detect minute initial neoplastic lesions. After finding those lesions, immunofluorescence staining for other markers was performed on serial sections as explained below in detail.

Histopathological changes were observed in hematoxylin and eosin (H&E)-stained specimens on the same sections after immunofluorescence staining. Colorectal neoplasms were diagnosed based on Ward’s description [42]. Uni-, bi-, or tri-cryptal neoplastic (dysplasia) and micro-neoplastic lesions (less than 300 μm wide) could be also observed in these sections.

### 4.5. Domain-Specific Antibodies against the Phosphorylated Smad2 and Smad3

Rabbit polyclonal anti-human pSmad2/3L-Thr (Smad2: Thr 220, Smad3: Thr 179) sera were raised against the phosphorylated linker region of Smad2 and Smad3 by immunizing rabbits with synthetic peptides [24,43,44]. The antisera were subjected to antigen affinity purification using phosphorylated peptides as described previously [45].

### 4.6. Immunohistochemistry

Immunofluorescence staining was performed on the formalin-fixed paraffin-embedded sections as previously described [27,28,37]. The paraffin-embedded sections were deparaffinized, washed with xylene and ethanol, and rehydrated. Non-enzymatic antigen retrieval was performed by heating the sections at 121 °C for 10 min in 0.01 M sodium citrate buffer (pH 6.0). After cooling, the sections were immersed in Tris-buffered saline (TBS) and blocked with 3% bovine serum albumin in TBS for 5 min. The primary antibodies (Abs) were diluted with TBS containing 0.1% Tween 20 and incubated at 4 °C in a humidity chamber. The primary Abs used in this study were as follows: mouse monoclonal anti-human β-catenin Ab (sc-7963, Santa Cruz Biotechnology, Santa Cruz, CA, USA), rat monoclonal anti-mouse Ki67 Ab (652402, BioLegend, San Diego, CA, USA), mouse monoclonal anti-human CDK4 Ab (sc-23896, Santa Cruz Biotechnology), rabbit monoclonal anti-human Sox9 Ab (ab185230, Abcam, Cambridge, UK), goat polyclonal anti-human B cell-specific Moloney murine leukemia virus integration site 1 (Bmi1) Ab (ab115251, Abcam), rabbit monoclonal anti-mouse cyclin D1 Ab (ab16663, Abcam), and rabbit polyclonal anti-human pSmad2/3L-Thr Ab. The secondary Abs used were the appropriate species-specific AlexaFluor (488 or 568)-conjugated Abs (Thermo Fisher Scientific, Waltham, MA, USA). Slides were mounted in VECTASHIELD mounting medium containing 4′,6-diamidino-2-phenylindole (DAPI) (Vector Laboratories, Burlingame, CA, USA) and stained for the nuclei. Images were captured using a fluorescence microscope (Olympus, Tokyo, Japan). After immunofluorescence staining, the specimen slides were immersed in distilled water and the cover glasses were gently removed to avoid tissue damage. After extensive soaking in TBS, H&E staining was performed using a standard staining procedure. Following this, the same sections were observed under a light microscope.

Well-oriented lesions from the base to the surface were selected to measure the locations of the lesions and immunostaining-positive cells within the mucosae using the inForm software (PerkinElmer, Waltham, MA, USA) according to the manufacturer’s instructions. The mucosae were vertically divided into three sections of the same length, and lesions and immunostaining-positive cells were confirmed to be present at the base, center, or top of them.

### 4.7. Statistical Analysis

Values are expressed as the mean ± standard error of the mean. Data were analyzed using a one-way analysis of variance followed by Fisher’s protected least significant difference test.

*p* < 0.05 was indicative of statistical significance.

## Data Availability

The datasets used and/or analyzed during the current study are available from the corresponding author upon reasonable request.
